# Predicting micropapillary or solid pattern of lung adenocarcinoma with CT-based radiomics, conventional radiographic and clinical features

**DOI:** 10.1186/s12931-023-02592-2

**Published:** 2023-11-14

**Authors:** Zhe Wang, Ning Zhang, Junhong Liu, Junfeng Liu

**Affiliations:** 1 Hebei Medical University Fourth Hospital, Thoracic Surgery. 12 Jiankang Road, Shijiazhuang, China; 2 Department of Radiology, Hebei Medical University Fourth Hospital, 12 Jiankang Road, Shijiazhuang, China

**Keywords:** Lung adenocarcinoma, Radiomics, Prediction

## Abstract

**Background:**

To build prediction models with radiomics features, clinical/conventional radiographic signs and combined scores for the discrimination of micropapillary or solid subtypes (high-risk subtypes) of lung adenocarcinoma.

**Methods:**

This retrospective study enrolled 351 patients with and without high-risk subtypes. Least Absolute Shrinkage and Selection Operator (LASSO) regression with cross-validation was performed to determine the optimal features of radiomics model. Missing clinical data were imputed by Multiple Imputation with Chain Equations (MICE). Clinical model with radiographic signs was built and scores of both models were integrated to establish combined model. Receiver operating characteristics (ROC) curves, area under ROC curves and decision curve analysis (DCA) were plotted to evaluate the model performance and clinical application.

**Results:**

Stratified splitting allocated 246 patients into training set. MICE for missing values obtained complete and unbiased data for the following analysis. Ninety radiomic features and four clinical/conventional radiographic signs were used to predict the high-risk subtypes. The radiomic model, clinical model and combined model achieved AUCs of 0.863 (95%CI: 0.817–0.909), 0.771 (95%CI: 0.713–0.713) and 0.872 (95%CI: 0.829–0.916) in the training set, and 0.849 (95%CI: 0.774–0.924), 0.778 (95%CI: 0.687–0.868) and 0.853 (95%CI: 0.782–0.925) in the test set. Decision curve showed that the radiomic and combined models were more clinically useful when the threshold reached 37.5%.

**Conclusions:**

Radiomics features could facilitate the prediction of subtypes of lung adenocarcinoma. A simple combination of radiomics and clinical scores generated a robust model with high performance for the discrimination of micropapillary or solid subtype of lung adenocarcinoma.

**Supplementary Information:**

The online version contains supplementary material available at 10.1186/s12931-023-02592-2.

## Background

Lung adenocarcinoma is the most common type of lung cancer [1]. According to the new IASLC/ATS/ERS Lung Adenocarcinoma Classification, it is classified as six subtypes: lepidic, acinar, papillary, micropapillary, solid and invasive mucinous [2, 3]. Among them, micropapillary and solid pattern showed distinctively worse prognosis than others [4, 5]. Reports revealed that the 5-year overall survival for micropapillary or solid pattern presented in lung adenocarcinoma was 67% in stage IA patients, while that of non- micropapillary or solid pattern patients could reach 94% [6]. Thus, micropapillary and solid subtypes are often classified as “high-risk” patterns, and worth further investigation [7].

Surgery for early-stage lung cancer is resecting “as less lung tissue as possible”. For small-sized, peripheral lung cancer, researchers recommend segmentectomy or wedge resection instead of lobectomy as standard surgical treatment [8, 9]. However, these sub-lobar resection seems to be insufficient for micropapillary and solid subtypes [10], as regional and mediastinal lymph nodes metastasis, blood vessel invasion and STAS (Spread Through Air Space) are often observed in such subtypes [11]. This is particularly confusing in bilateral lung lesion patients, who often receive sub-lobectomy to preserve more pulmonary function. Insufficient resection for high-risk lesion brings higher recurrence and worse survival, which cancel-out the benefit from sub-lobectomy. Hence, extensive resection plus systematic mediastinal may still be necessary for them, thereby raising the need for preoperatively diagnosis of the micropapillary or solid components. In addition, no lung adenocarcinoma subtype diagnosis can be made from preoperative or intro-operative biopsy. High-risk subtypes are determined only from paraffin embedding tissue pathology examination, which is often 7 days after surgery. Currently, there is a lack of investigation on the relationship between clinical and radiographic signs and high-risk subtypes of lung adenocarcinoma. Some study might show that tumor size, solid mass and maximal standardized uptake value could possibly be independent predictors for the two high-risk subtypes [5]. However, a validated predicting model is yet to be determined.

Radiomics is emerging as a novel quantitative analysis with abundant features extracted from CT images and served as “big data” in further machine learning [12]. It has been widely engaged in the prediction for the differential of benign and malignant tumour, survival of cancer and treatment reaction of immune checkpoints [13–15]. For example, Kinahan et al. compared the diagnosis of lung cancer with semantic and quantitative texture features in 238 individuals. They achieved an AUC of 0.85 to 0.88 with these radiomics features [16]. In addition, Tian and his colleagues incorporated radiomics features to build prediction model for the epidermal growth factor receptor (EGFR) mutation status in lung cancer. Their AUCs for the training and validation cohort were 0.8618 and 0.8725, demonstrating the excellent capability of radiomics features in lung cancer prediction [17]. A recent systematic review and meta-analysis also showed that radiomics signatures combined with deep learning algorithms could serve as a novel tool for the prediction of EGFR in non-small cell lung cancer [18]. These findings all strengthened the use of radiomics research in the field of cancer.

Thus, in our study, we aimed to establish a robust prediction model on the presence of micropapillary or solid pattern of lung adenocarcinoma based on the radiomics features. Moreover, we will also compare the prediction value of radiomics model with conventional clinical and radiographic variables.

## Methods

### Study cohort

**Fig. 1:**
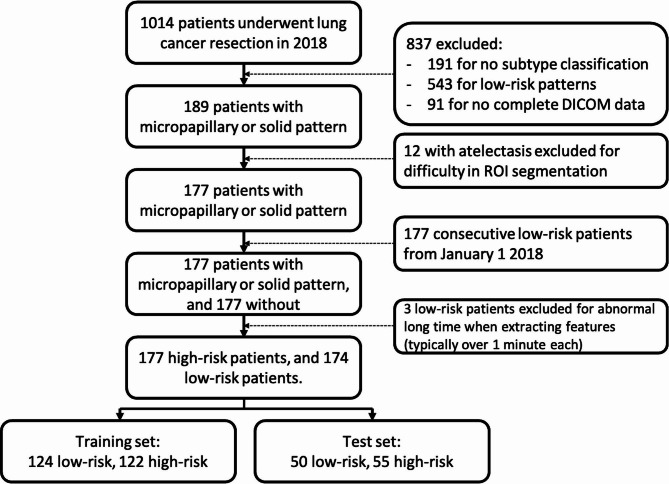
Flowchart shows the recruiting process of the study. Firstly, all high-risk patients in 2018 were selected, and then consecutive low-risk patients were enrolled to match the high-risk patients

This study was approved by the institutional ethnic committee and informed consent was waived due to the retrospective nature. Study procedure is demonstrated by the flowchart (Fig. 1).

**Fig. 2:**
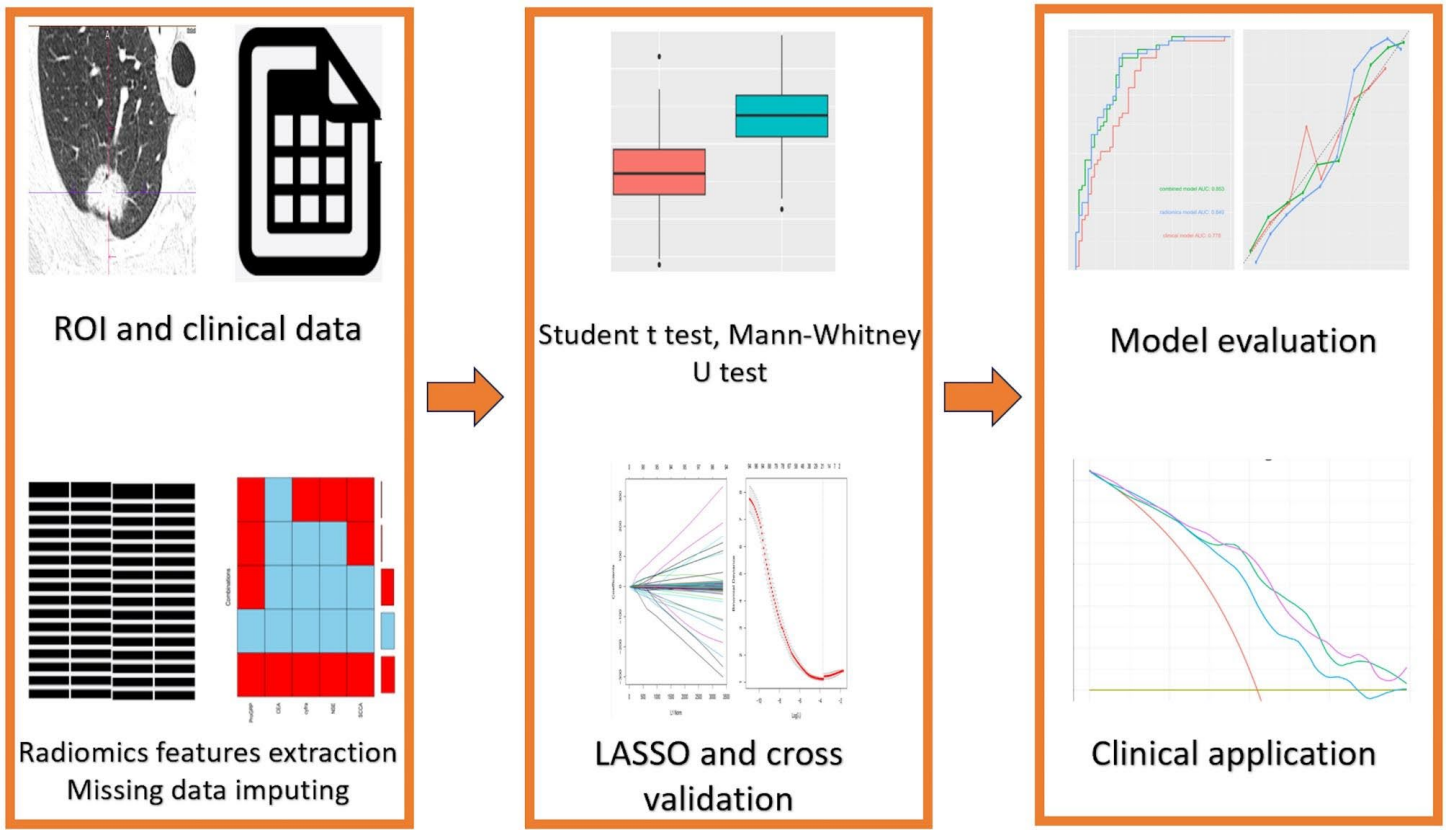
A general analysis process of the whole study

Patients diagnosed with lung adenocarcinoma from January 1 2018 to December 31 2018 were recruited firstly. Then the patients were selected according to the inclusion criteria as follows: (1) patients receiving surgical resection of the tumour; (2) pathological report including subtypes of adenocarcinoma; (3) patients with major clinical data; (4) patients CT images taken 2 weeks before surgery; (5) availability of CT images in DICOM. The exclusion criteria were as follows: (1) malignant tumour other than lung adenocarcinoma; (2) incomplete clinical and radiological data. In addition, to balance the sample size of high-risk group and low-risk group, patients of low-risk group were enrolled consequently until the sample size reach the high-risk group. We met unknow problems when extracting radiomics features from 3 patients in the low-risk group (extracting time was abnormally long). Finally, 351 patients were enrolled in this study. Patients were randomly allocated to the training set or test set. This was perfomed by the “createDataPartition” function in R package “caret”. This function creates indices of the training and test set according to the set ratio. The recruiting process was shown in Fig. 2.

### CT imaging

CT scanners used in the study were: SIEMENS SOMATOM Definition Flash, SIEMENS Sensation Open, GE MEDICAL SYSTEMS LightSpeed Pro 32, Philips iCT 256. During scanning, patients were instructed to hold their breath until the scanning finished. The scanning parameters were as follows: tube voltage 120 kV, tube current automatic, matrix 512*512, slice thickness 1, 1.25 or 1.5 mm. Contract enhanced chest CT was taken with a bolus dose of (70–90 mL) nonionic contrast agent iohexol or ioversol (300 mg·I/mL) injecting through the cubital vein by a high-pressure syringe with 20 s scan delay. The scanning range from the inlet of the thoracic cavity to the base of the lungs.

### Conventional radiological features collection, ROI segmentation and radiomics feature extraction

CT images were analyzed by a thoracic radiologist and a thoracic surgeon (NZ and ZW) with 8 and 10 years of experience respectively. Both doctors were blinded to the pathological report of the patients. Report on the CT images were performed independently by both doctors. Any discrepancies on the description and measurement of the lesion on the CT images were resolved through discussion. The following conventional features were reported: (1) nodule type: pure-ground glass opacity, sub-solid opacity with CTR (consolidation tumor ratio) less than 50%, sub-solid opacity with CTR (consolidation tumor ratio) more than 50%, pure solid nodule; (2) lobulated sign presented; (3) spiculated sign presented; (4) pleural traction presented; (5) bronchograms presented; (6) vessel retraction presented; (7) vacuole sign presented; (8) tumour edge smooth or blur; (9) adjacent to pleura (distance between tumour and pleural was less than 1 cm); (10) maximum tumour diameter.

The region of interest (ROI) was delineated by the two doctors independently without knowing the information of pathological results. Lung window was set to 1200 HU width and − 600 HU in level, while mediastinal window was 350 HU in width and 40 HU in level. Syngo.via platform (SIEMENS Heathineers, Erlangen Germany) was used for the ROI segmentation and radiomics features extraction. A built-in PyRadiomics based module in Syngo.via was used to extract the radiomics features. Extraction parameters following the PyRadimics package were also set before extraction to normalize the image heterogeneity: ResampledVoxelSize = 1*1*1, ResampledPixelSpacing = [1], interpolator: BSpine, filtering included wavelet, sqr, sqrt, log and exp. Feature types included GLDM, GLCM, Shape, First order, GLRLM, GLSZM, NGTDM. Bin width was set to 25. The interpretability and harmonization were tested by the PyRadiomics team to ensure the consistency from different imaging centers. Finally, a total of 1226 radiomic features were extracted.

### Clinical data collection and imputation

Electronic clinical records were reviewed, and characteristics relevant to the study were collected, including age, sex, smoking history, family cancer history, serum biomarker (ProGRP, CEA, cyfra, NSE, SCCA). These clinical variables were commonly selected for cancer risk factor analysis or prediction models based on researchers experience.

Missing data in serum biomarkers were analyzed and imputed by MICE package in R. Multiple Imputation by Chained Equations (MICE) is a stable, informative method that handle the missing data. The method uses a series of iterable prediction model to impute the missing data. For continues data like serum biomarker, MICE use PMM (predictive mean matching to impute the missing data). PMM builds prediction models with existing data and then predicts a series of data in the “missing data column”. Then it chooses a “predicted data” in the “prediction column” which is nearest to the “missing ones”. Finally, the real data in the “missing column” whose position is the same with the “predicted data” is used to fill the missing place.

### Radiomics features dimension reduction, model construction and evaluation

Several steps of radiomics features dimension reduction was performed to get the most predictable features. First, all the patients were randomly split into training and test set by a ratio of 7:3. All data were standardized by the scaling parameters of training set. Observer 1 (ZW) delineated all the patients ROI and observer 2 (NZ) repeated 50 of randomly selected patients. The interobserver consistency of radiomics feature extraction was assessed and features with intraclass correlation coefficient (ICC) over 0.75 were kept. Student t test and Mann-Whitney U test was then performed for each feature, according to the normality Shapiro test results. Features of values significantly different in two groups were selected with P value set to 0.05. Next, Spearman correlation analysis and Pearson correlation analysis were performed for non-normally and normally distributed features. Features with correlation coefficients over 0.9 were removed. Afterwards, least absolute shrinkage and selection operator (LASSO) were applied to training cohorts, with 10 folds cross validation tuning the optimal lambda. The lambda was decided when the mean square error of the prediction model reached one standard error, also known as “lambda.1se”. LASSO regression is an effective method for features reduction in high dimension data like radiomics study, while “lambda.1se” balanced the prediction value and number of features. Radscore was calculated by the features and coefficients selected by LASSO regression.

The radiomics and clinical model was established by logistic regression. As mentioned above, radiomics features selected by LASSO were used to build radiomics model. For clinical model, we performed univariate analysis first and clinical variables with P value less than 0.05 were included into multivariate logistics regression model to select the optimal clinical variables for logistics regression model. A cliscore was also calculated with this model for each patient. A combined dataset with radscore, cliscore and group labels was built for combined model construction using logistics regression. Logistic regression is one of a favorable algorithm in machine learning. Research compared logistic regression, random forest, and support vector machine classifiers in radiomics-based machine learning in different cancers. Logistic regression showed equivalent prediction values with others [19, 20]. Moreover, logistic regression is easier to interpret due to its close relationship with linear regression. In our study, we used logistic regression as the approach to build prediction models, and the same technique to validation the models.

The receiver operating characteristic (ROC) curve and area under the ROC curve (AUC) were used as main methods to evaluate the performance of the three models. Other model evaluation indicators including accuracy, sensitivity, specificity, positive predictive value, and negative predictive value. DeLong test and Bland-Altman analysis were used to compare the ROC curves. Calibration curves were used to visualize the Hosmer-Lemeshow test for the logistic regression. Decision curves were used to evaluate the clinical significance of the models.

### Statistical analysis

All statistical analyses were performed using R (version 4.4.2, R foundation, Viena, Austria). Major packages used in this study included ‘caret’ (version 6.0), ‘glmnet’ (version 4.1), ‘glm’ function in ‘stats’ (version 4.3.0), ‘pROC’ (version 1.18.4), ‘blandr’ (version 0.5.1), ‘tidyverse’ (version 2.0.0), ‘mice’ (version 3.16.0). Continuous variables were presented as mean ± standard deviation, while categorical variables were presented as count numbers. Shapiro test was used for the normality test. For varaibles normally distributed, Student t test was used to compare the statistical difference. Other variables were compared by Mann-Whitney U test. Significance level was set to 0.05, two sided.

## Results

### Clinical and conventional radiographic characteristics, with missing data imputation

A total of 351 patients were included in this analysis. Among them, 246 were randomly split into training set and 105 in test set. Clinical and conventional radiographic characteristics of baseline between training and test set were shown in Table 1. There were no significantly different clinical and conventional radiographic features between the two sets. Training set was then used for data analysis and model building.

**Table 1:** clinical characteristics of training and test set at baseline

Characteristic	test, N = 105^1^	train, N = 246^1^	p-value^2^
age	63 (56, 67)	62 (55, 67)	0.14
gender			> 0.9
F	53 (50%)	123 (50%)	
M	52 (50%)	123 (50%)	
max diameter(mm)	37 (24, 50)	36 (25, 48)	0.5
smoking history	40 (38%)	89 (36%)	0.7
lung cancer family history	12 (11%)	24 (9.8%)	0.6
ProGRP	44 (37, 56)	43 (38, 56)	> 0.9
CEA	3 (2, 6)	3 (2, 6)	0.7
cyfra	2.20 (1.70, 3.40)	2.20 (1.70, 3.38)	0.9
NSE	13.7 (12.4, 15.3)	13.3 (12.2, 15.1)	0.3
SCCA	0.80 (0.60, 0.90)	0.80 (0.60, 0.98)	> 0.9
type			0.6
CTR < 0.5	11 (10%)	20 (8.1%)	
CTR > 0.5	5 (4.8%)	6 (2.4%)	
pure-GGO	5 (4.8%)	14 (5.7%)	
solid	84 (80%)	206 (84%)	
lobular	42 (40%)	92 (37%)	0.6
spiculation	52 (50%)	149 (61%)	0.055
pleural traction	27 (26%)	63 (26%)	> 0.9
air bronchus	21 (20%)	54 (22%)	0.7
vessel	43 (41%)	104 (42%)	0.8
hollow	17 (16%)	33 (13%)	0.5
smooth edge	6 (5.7%)	13 (5.3%)	0.9
adjacent to pleural	15 (14%)	30 (12%)	0.6

^1^Median (IQR); n (%)

^2^Wilcoxon rank sum test; Pearson’s Chi-squared test; Fisher’s exact test

**Fig. 3:**
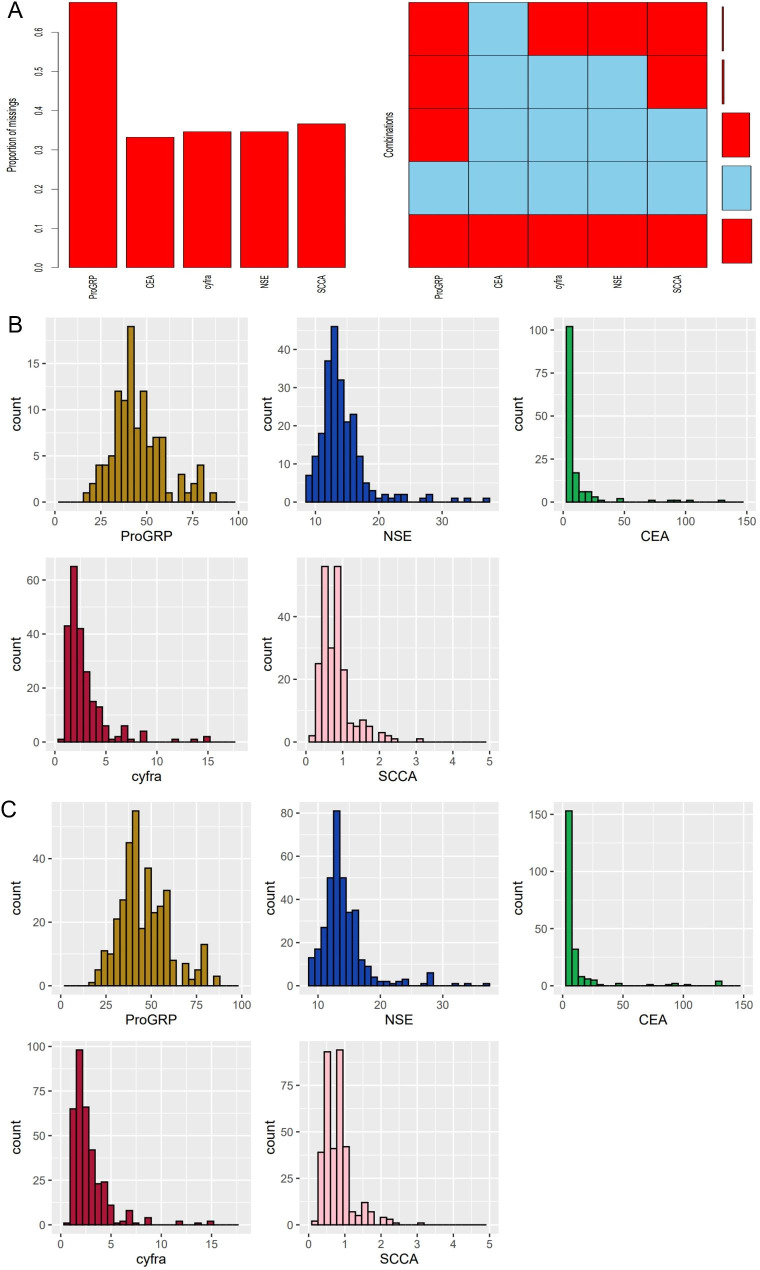
Missing values evaluation and imputation by MICE. (**A**) demonstrated missing value distribution among five clinical variables. (**B**) and (**C**) demonstrated the data distribution before and after MICE. MICE: multivariate imputation by chained equations

The missing value distribution in the lung cancer biomarker was shown in Fig. 3 (before and after mice and missing). Multiple Imputation by Chained Equations was adopted to impute the missing values while keep the original data distribution, which make the statistical analysis practicable as well as the results convincible.

Out of the 246 patients in the training set, there were significantly more male in high-risk group than the low-risk group (61% vs40%, p < 0.001). Moreover, lesion size was larger in the high-risk group (39 vs. 32, p < 0.001). Solid lesion is also the predominant type in high-risk group (98% vs. 69%, p < 0.001). Univariate analysis also showed that high-risk group had less signs of air bronchus (16% vs. 28%, p = 0.017), vessel in the lesion (32% vs. 52%, p = 0.001), and lobular (30% vs. 45%, p = 0.011). Furthermore, smoking history was much more often in high-risk group as well (47% vs. 26, p < 0.001). In multivariate analysis, diameter max, type, air bronchus and lobular sign were shown to be significantly different between two groups. Full univariate and multivariate results were shown in Table 2.

**Table 2:** clinical characteristics of high-risk and low-risk groups in the training set

Characteristic	high risk, N = 122^1^	low risk, N = 124^1^	p-value^2^	multivariate
age	62 (55, 67)	61 (55, 67)	> 0.9	
gender			< 0.001	
Female	48 (39%)	75 (60%)		
Male	74 (61%)	49 (40%)		
max diameter(mm)	39 (28, 54)	32 (22, 43)	< 0.001	0.008
smoking history	57 (47%)	32 (26%)	< 0.001	
lung cancer family history	12 (9.8%)	12 (9.7%)	> 0.9	
ProGRP	42 (36, 54)	44 (39, 58)	0.11	
CEA	4 (2, 7)	3 (2, 5)	0.2	
cyfra	2.25 (1.70, 3.48)	2.20 (1.68, 3.30)	0.7	
NSE	13.50 (12.60, 15.80)	13.11 (12.10, 14.50)	0.062	
SCCA	0.80 (0.60, 1.00)	0.70 (0.50, 0.90)	0.2	
type			< 0.001	< 0.001 (pure-GGO)
CTR < 0.5	1 (0.8%)	19 (15%)		
CTR > 0.5	1 (0.8%)	5 (4.0%)		
pure-GGO	0 (0%)	14 (11%)		
solid	120 (98%)	86 (69%)		
lobular	36 (30%)	56 (45%)	0.011	0.013
spiculation	76 (62%)	73 (59%)	0.6	
pleural traction	37 (30%)	26 (21%)	0.093	
air bronchus	19 (16%)	35 (28%)	0.017	0.011
vessel	39 (32%)	65 (52%)	0.001	
hollow	17 (14%)	16 (13%)	0.8	
smooth edge	9 (7.4%)	4 (3.2%)	0.15	
adjacent to pleural	15 (12%)	15 (12%)	> 0.9	

^1^Median (IQR); n (%)

^2^Wilcoxon rank sum test; Pearson’s Chi-squared test; Fisher’s exact test

### Radiomics feature selection and prediction models building

**Fig. 4:**
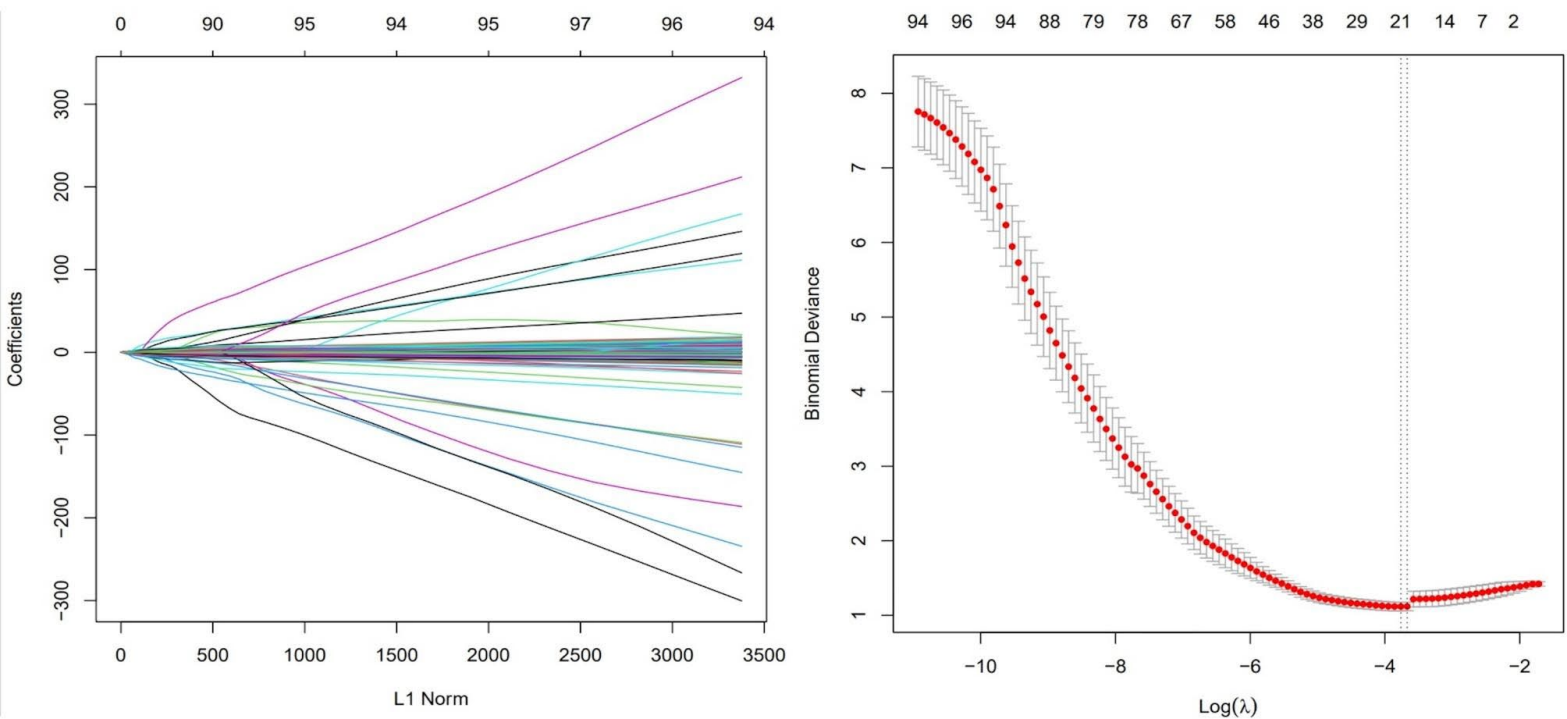
LASSO regression and cross validation

After a series of preprocessing including interobserver analysis, Student t test, Mann-Whitney U test and correlation analysis, a total of 116 radiomics features were filtered from the overall 1226 features extracted from ROIs. Then LASSO regression with 10-fold cross validation was used for dimension reduction, with lambda set to one-standard-error of model mean square error (MSE). Ninety features were finally selected and radiomics prediction model was then established. Radscore was calculated by multiply the value of each feature with their coefficients. This process results were shown in Fig. 4; Table 3.

**Table 3:** radiomic features selected by LASSO regression and their coefficiencies

Coefficients	Feature family	Feature subtype
-0.01469447		Intercept
0.23464576	original	shape_Flatness
-0.07955321	square	glcm_InverseVariance
-0.23345119		glrlm_ShortRunLowGrayLevelEmphasis
0.11685419	exponential	firstorder_90Percentile
0.27726069		firstorder_Mean
0.06374112		firstorder_TotalEnergy
0.01856495		glszm_SizeZoneNonUniformityNormalized
-0.01994271	wavelet	HHH_firstorder_Kurtosis
0.17396774		HHH_glcm_Idmn
-0.13376102		HLH_glcm_Idn
0.01637181		HLL_firstorder_Skewness
0.23530962		HLL_glszm_ZoneEntropy
0.15230831		LHH_glcm_MCC
0.13845049		LHL_glszm_SmallAreaLowGrayLevelEmphasis
-0.13695941		LLH_glcm_MCC
0.09393939		LLH_glszm_GrayLevelNonUniformityNormalized
0.16229105		LLL_firstorder_90Percentile
0.36867058		LLL_firstorder_Kurtosis
0.40500583		LLL_glcm_JointEntropy

**Fig. 5:**
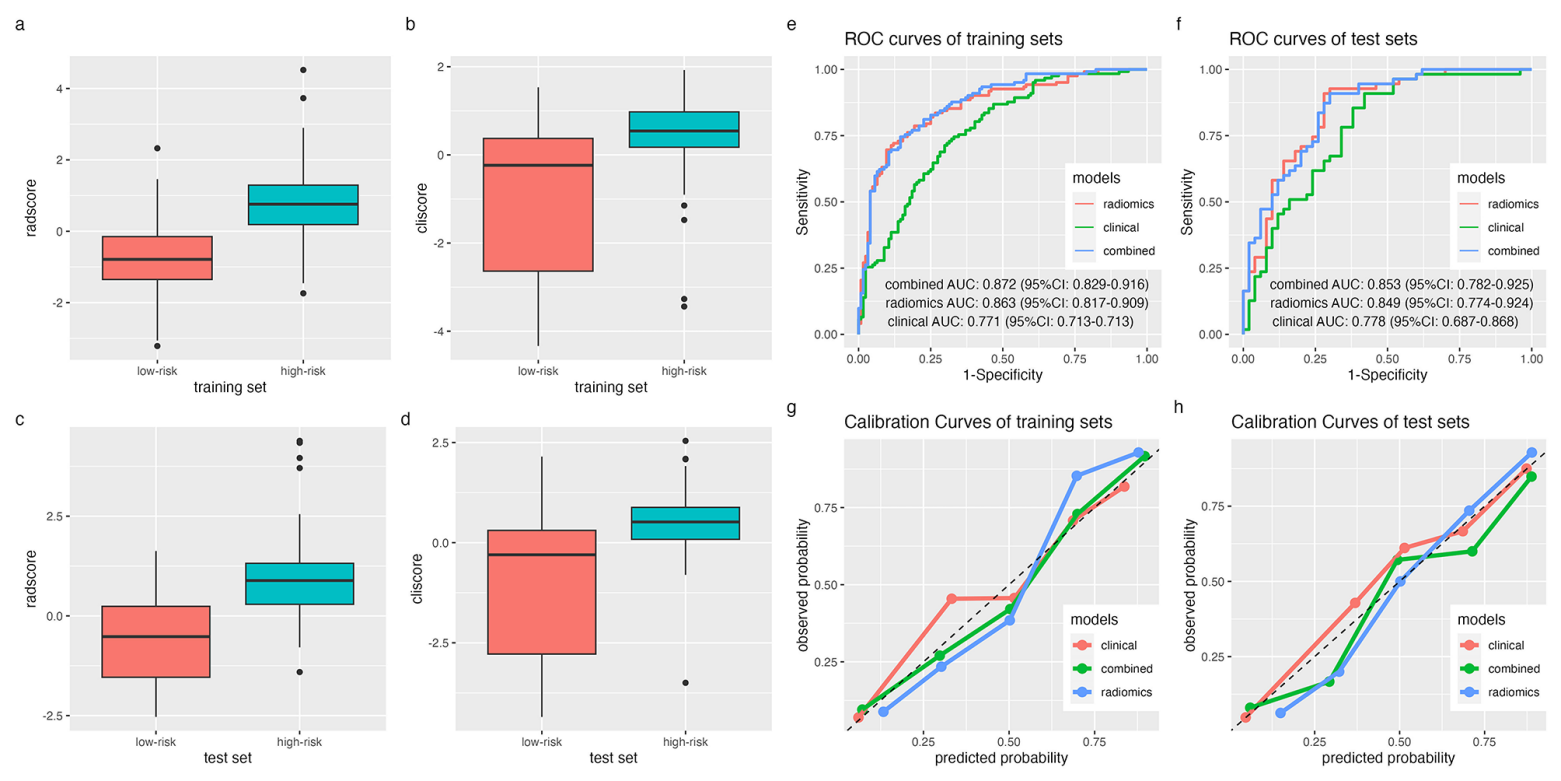
Radscores and cliscores in training (**a,b**) and test (**c,d**) set. ROC curves (**e,f**) and calibration curves (**g,h**) of three models. In ROC curves, higher AUC indicated better capability of discrimination. In calibration curves, being closer to the 45-degree black dotted line indicated better model fitting

Clinical prediction model was built with the four clinical-conventional radiographic characteristics by logistic regression (supplementary file 1). Characteristics value was set to 1 when presented and 0 when absent. Afterwards a “cliscore” (indicating “clinical score”) was also calculated by multiplying the values and corresponding coefficients. The radscore and cliscore of each patient were gathered to build new training set and test set, in which a combined model was build. Figure 5 (a-d) showed the scores between high-risk and low-risk groups in the training and test set.

### Model evaluation and clinical significance

**Fig. 6:**
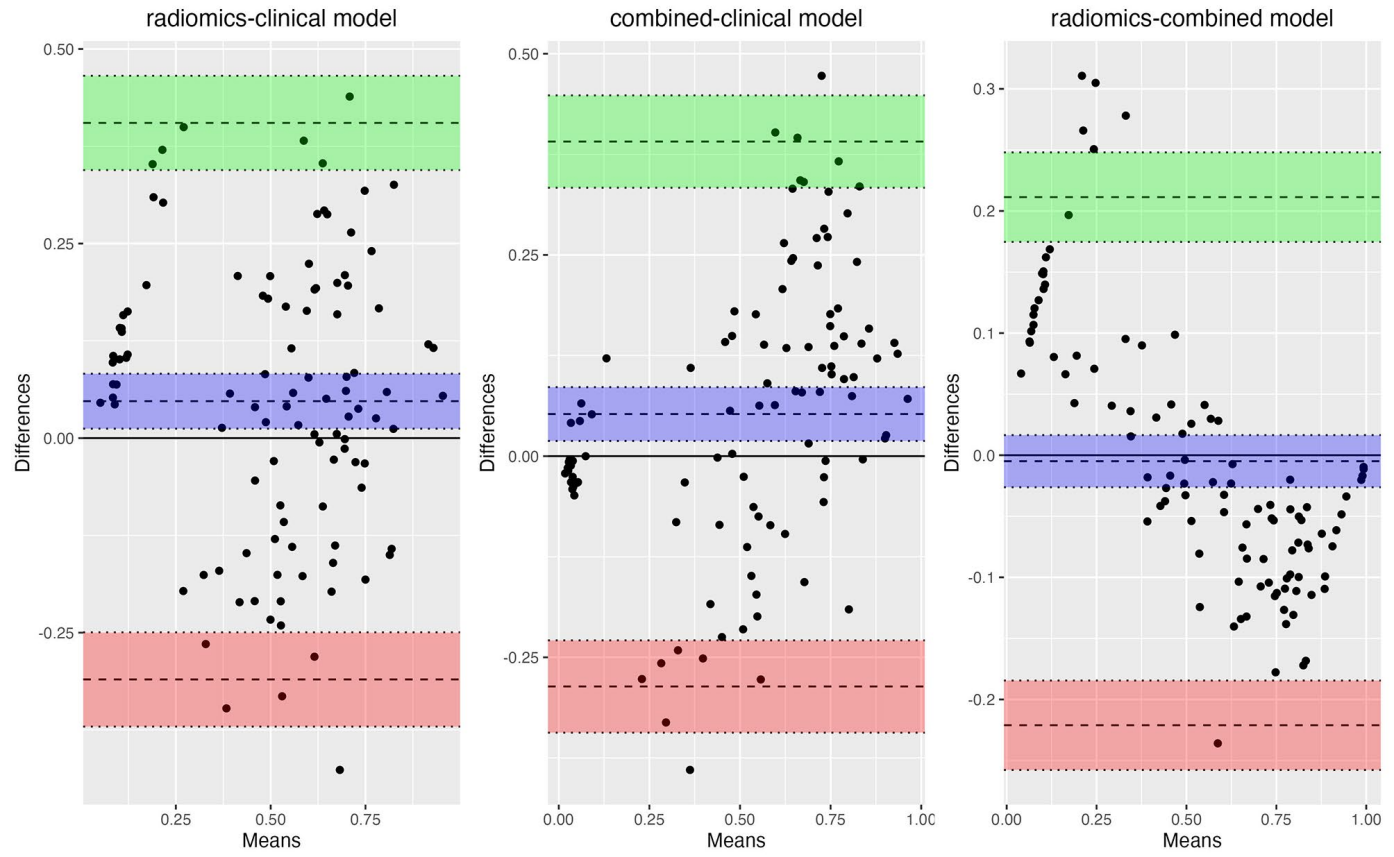
Bland-Altman analysis for the comparation of the models

The radiomics model, clinical model and combined model were built with the training set and validated by the test set. ROC curves and its AUCs showed that radiomics model and combined model had good predicting value (AUCs: 0.849, 95%CI: 0.774–0.924 for radiomics model and 0.853, 95%CI: 0.782–0.925 for combined model in test set), while clinical model also had acceptable prediction value with AUC of 0.778 (95%CI: 0.687–0.868) in test set (Fig. 5e,f). DeLong test revealed that there was significant difference between ROCs of radiomics model and clinical model (p = 0.003), combined model and clinical model (p < 0.001), whereas the combined model did not show better prediction ability than radiomics model (p = 0.125). Bland-Altman analysis also showed similar results, with p values of 0.009, 0.002 and 0.651 for the comparation of radiomics-clinical model, combined-clinical model and radiomics-combined model (Fig. 6). Detailed model estimators demonstrated in Table 4.

**Table 4:** model evaluation measurements

Name	AUC	AUC_upper95%CI	AUC_lower95%CI	Accuracy	Kappa	Sensitivity	Specificity	Pos_Pred_Value	Neg_Pred_Value	F1
rad_train	0.863	0.817	0.909	0.785	0.569	0.782	0.787	0.789	0.780	0.785
rad_test	0.849	0.774	0.924	0.762	0.521	0.720	0.800	0.766	0.759	0.742
cli_train	0.771	0.713	0.713	0.695	0.391	0.597	0.795	0.747	0.660	0.664
cli_test	0.778	0.687	0.868	0.705	0.404	0.620	0.782	0.721	0.694	0.667
comb_train	0.872	0.829	0.916	0.785	0.569	0.758	0.811	0.803	0.767	0.780
comb_test	0.853	0.782	0.925	0.790	0.577	0.700	0.873	0.833	0.762	0.761

**Fig. 7:**
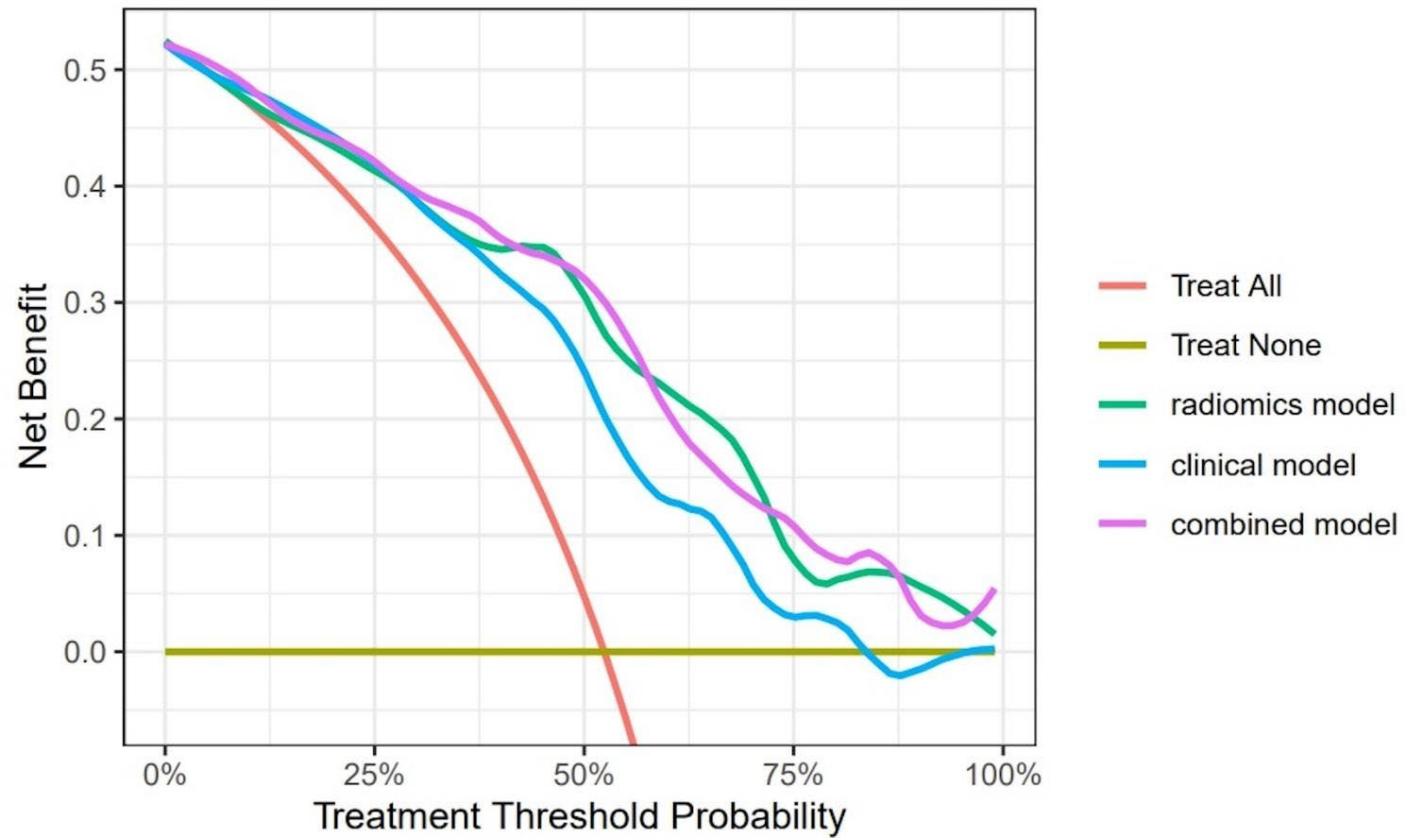
Decision curves for the three models. Models further from the ‘Treat All’ and ‘Treat None’ are better in clinical benefit

In calibration curve analysis, the three models were proved to fit well with the training set, while also reasonably fitted in the test set (Fig. 5g,h). Hosmer-Lemeshow test indicated no significant difference in the three models in training and test set (Training set: p = 0.193, 0.822 and 0.741 for radiomics, clinical and combine model. Test set: p = 0.13, 0.78, 0.81 for radiomics, clinical and combine model). Decision curve analysis assess the clinical usage of a prediction model by calculating the net benefit of treatment with different thresholds and compare it with “treat all” and “treat none”. Figure 7 demonstrated that the radiomics and combined model started to practice better clinically than the clinical model when the threshold reached 37.5%.

## Discussion

The histological subtype diagnosis of lung adenocarcinoma is still based on the tumour resection or biopsy [21]. However, biopsy sometimes cannot provide evidence strong enough for subtype classification [22, 23]. New non-invasive method is required for preoperative prediction for the high-risk subtypes. In this study, clinical with conventional radiographic features, and radiomics features have been incorporated into prediction models for the present of micropapillary or solid pattern in lung adenocarcinoma. Different from other combined models on lung adenocarcinoma prediction, which enrolled a bunch of radiomics and clinical features, we creatively built a simple combined model with “radscore” and “cliscore” calculated by the radiomics and clinical model. The performance of three models was compared and their clinical use was evaluated. Our results showed that radiomics and combined model performed better than traditional clinical and radiographic variables, suggesting that it could be potentially employed in the preoperative classification of subtypes of lung adenocarcinoma. This finding would further remind surgeons about high-risk lesions before planning surgery for lung adenocarcinoma, even though the lesions may be small and just “ordinary”.

Micropapillary or solid subtypes of lung adenocarcinoma have significantly worse survival than other subtypes [24]. However, lung resection for small lesions and bilateral lesions were normally sub-lobectomies to keep as much lung tissue as possible, while might be not enough for micropapillary or solid subtypes [10]. This was even more crucial for patients with bilateral nodules, as lobectomy and sub-lobectomy were planned carefully before surgery. However, lung adenocarcinoma subtype diagnosis is often made 7 days after surgery with the paraffin embedded pathology examination. There is no appropriate additional treatment when high-risk pattern is identified, and the resection is sublobectomy. Some studies have focused on the classification for the subtypes of lung adenocarcinoma, but with their own limitations. He et al. obtained a highest AUC of 0.73 on the test set among all the models, while Li et al. reported an excellent AUC of 0.91, but both studies had no clinical features integrated. Xu and his colleagues only identified micropapillary pattern and no clinical model built either. Yang et al. achieved accuracy rates of 84.2% and 91.6% in the prediction models, whereas the sample size was no more than 100 [24–26]. We built three models to predict the high-risk subtypes preoperatively. Clinical model with conventional features showed moderate predictive value for the discrimination of high and low risk type. The multivariate logistic regression model showed that max diameter of lesion, lobulation and solid type all contributed to the differential diagnosis, which is consistent with finding of Yuan et al [27]. Additionally, Seo also proposed that solid subtypes were likely to have larger diameter and appear as solid morphology, and tumours with spiculation or lobulation were prone to low risk subtypes [28–30]. These findings were also confirmed by our multivariate analysis.

However, clinical model was not robust enough in predicting high-risk subtypes of lung adenocarcinoma, with relatively low sensitivity, specificity, and accuracy. In our study, the radiomics model showed better predictive performance over clinical model, similar to combined model, indicating that the clinical features with conventional radiographic signs did not have strong predictive value on the classification purpose. The radiomics features selected generally described the heterogeneity of grey levels and density of the tumour, which is in accordance with some radiographic features such as tumour type (solid or sub-solid) and air bronchus, and they somehow mutually confirmed each other [31]. Therefore, we hypothesised that the radiomics features which reflected the grey level and density could be further investigated in differentiating subtypes of lung adenocarcinoma. In addition to radiomics features on the grey level and density, some researchers have validated that specific radiomics features could be related to clinical prognosis like overall survival among multi-organ cancer. Nguyen Quoc Khanh Le et al. demonstrated that a set of radiomics features extracted from CT images of lung cancer were evaluated in lung, head and neck, and kidney data, showing improved time-dependent AUC of 0.736 (95% CI 0.654, 0.819), 0.732 (95% CI 0.655, 0.809), and 0.834 (95% CI 0.722, 0.946) [32]. This finding reminded us that we may put an eye on “function-specific feature selection” when selecting radiomics features.

Multiple imputation by chain equation is an advanced and widely accepted technique for missing data manipulating. The statistical theory is based on regression models, which is built from existing data and then predicts the missing values. Multiple imputation by chain equation is typically performed by MICE package in R [33]. In this process, a new column is calculated with suffix “imp” for the column with missing data. Then whether each missing value is closer to the previous or the next value is decided by the sequence of the newly calculated column. Finally, the missing value is imputed by the closer value in its own column. In our study, the missing data were mainly from the serum biomarker of carcinoembryonic antigen (CEA), nerve-specific enolase (NSE), cytokeratin 19 fragment (CYFRA21-1), squamous cell carcinoma antigen (SCC-Ag), and pro-gastrin-releasing peptide (ProGRP), due to different clinical practise of patients. Removing patients with missing data would lead to markedly reduce in sample size, while introducing MICE could preserve the sample size and keep statistics working. Although univariate results showed that no biomarker was expressed differently between two groups. Serum biomarker of CEA, NSE, CYFRA21-1, SCC-Ag, and ProGRP has been investigated and applicated in clinical practise. However, their value of differentiation diagnosis mainly focusses on the major types of non-small cell lung cancer and small lung cancer, whether they could be used to discriminate the subtypes of lung adenocarcinoma remains uncertain [34, 35]. To further predict the subtypes of adenocarcinoma, novel biomarkers such as extracellular vesical associated microRNA, and radiomics biomarkers shown in our study, may be adopted.

LASSO regression is popular in dimension reduction and feature selection for “big data”. Almost every radiomics study adopted cross validation of LASSO regression as a main approach in the data processing. However, concerns have been raise that randomness exists in the cross validation, which lead to inconsistent optimal values of lambda [36]. During our analysis, we also experienced time consuming process of lambda optimization. In addition, training and test set splitting also brought instability. The calibration curve of clinical model fitted even better than the other two models, probably causing by the splitting issue. To solve these problems, scientists are working on updated dimension reduction methods with LASSO regression. For example, Damian and Geroge et al. proposed a “StaVarSel” method using nested cross validation combined with frequency selection by LASSO [37]. They achieved 100% specificity and 95.2% sensitivity with Stabilised nested cross validation compared with standard nested cross validation (66.7% in specificity and 47.1% in sensitivity). This method may be promising in radiomics application with reasonable revision.

This study, nevertheless, has certain limitations. First, this was a retrospective study from a single centre, with inevitable selection bias and other confounding factors. A multi-centre prospective study with larger population is required in further analysis. Second, as a single centre study, there was no external validation set recruited. The model would be more convincible if validated in independent external validation set. Third, study data was consisted of radiomic features extracted from various scanners. Though 1*1*1 mm voxel isotropic resampling was applied, a single scanner data analysis would be conducted in future investigation.

## Conclusions

In conclusion, our study revealed that radiomics features by themselves could facilitate the prediction of subtypes of lung adenocarcinoma. Additionally, clinical missing data could be imputed by MICE and then be used to calculate clinical scores, forming a simple but discriminative dataset with radiomic scores. Radiomics and combined models had reasonable prediction value for micropapillary or solid subtype of lung adenocarcinoma.

### Electronic supplementary material

Below is the link to the electronic supplementary material.

Supplementary Material 1

## Data Availability

The datasets used and/or analysed during the current study are available from the corresponding author on reasonable request.

## Abbreviations

LASSO: Least Absolute Shrinkage and Selection Operator
ROC: Receiver operating characteristics
AUC: Area under ROC curves
STAS: Spread Through Air Space
MICE: Multiple Imputation with Chain Equations
MSE: Mean Square Error

## References

[1] Li C, Lu H (2018). Adenosquamous carcinoma of the lung. OncoTargets Ther.

[2] Eguchi T, Kadota K, Park BJ, Travis WD, Jones DR, Adusumilli PS (2014). The new IASLC-ATS-ERS lung adenocarcinoma classification: what the surgeon should know. Semin Thorac Cardiovasc Surg.

[3] Travis WD, Brambilla E, Noguchi M, Nicholson AG, Geisinger KR, Yatabe Y (2011). International Association for the Study of Lung Cancer/American Thoracic Society/European Respiratory Society International Multidisciplinary Classification of Lung Adenocarcinoma. J Thorac Oncol off Publ Int Assoc Study Lung Cancer.

[4] Xu L, Zhou H, Wang G, Huang Z, Xiong R, Sun X (2022). The prognostic influence of histological subtypes of micropapillary tumors on patients with lung adenocarcinoma ≤ 2 cm. Front Oncol.

[5] Cha MJ, Lee HY, Lee KS, Jeong JY, Han J, Shim YM (2014). Micropapillary and solid subtypes of invasive lung adenocarcinoma: clinical predictors of histopathology and outcome. J Thorac Cardiovasc Surg.

[6] Tsao M-S, Marguet S, Le Teuff G, Lantuejoul S, Shepherd FA, Seymour L (2015). Subtype classification of lung Adenocarcinoma Predicts Benefit from Adjuvant Chemotherapy in patients undergoing complete resection. J Clin Oncol off J Am Soc Clin Oncol.

[7] Peng B, Li G, Guo Y (2021). Prognostic significance of micropapillary and solid patterns in stage IA lung adenocarcinoma. Am J Transl Res.

[8] Saji H, Okada M, Tsuboi M, Nakajima R, Suzuki K, Aokage K (2022). Segmentectomy versus lobectomy in small-sized peripheral non-small-cell Lung cancer (JCOG0802/WJOG4607L): a multicentre, open-label, phase 3, randomised, controlled, non-inferiority trial. Lancet Lond Engl.

[9] Suzuki K, Watanabe S, Wakabayashi M, Moriya Y, Yoshino I, Tsuboi M (2017). A nonrandomized confirmatory phase III study of sublobar surgical resection for peripheral ground glass opacity dominant Lung cancer defined with thoracic thin-section computed tomography (JCOG0804/WJOG4507L). J Clin Oncol.

[10] Nitadori J, Bograd AJ, Kadota K, Sima CS, Rizk NP, Morales EA (2013). Impact of micropapillary histologic subtype in selecting limited resection vs lobectomy for lung adenocarcinoma of 2 cm or smaller. J Natl Cancer Inst.

[11] Mino-Kenudson M (2020). Significance of Tumor spread through air spaces (STAS) in Lung cancer from the pathologist perspective. Transl Lung Cancer Res.

[12] Lambin P, Rios-Velazquez E, Leijenaar R, Carvalho S, van Stiphout RGPM, Granton P (2012). Radiomics: extracting more information from medical images using advanced feature analysis. Eur J Cancer Oxf Engl 1990.

[13] El Ayachy R, Giraud N, Giraud P, Durdux C, Giraud P, Burgun A et al. The Role of Radiomics in Lung Cancer: From Screening to Treatment and Follow-Up. Front Oncol [Internet]. 2021 [cited 2023 Jul 2];11. Available from: https://www.frontiersin.org/articles/10.3389/fonc.2021.603595. 10.3389/fonc.2021.603595 PMC8131863 34026602

[14] Walls GM, Osman SOS, Brown KH, Butterworth KT, Hanna GG, Hounsell AR (2022). Radiomics for Predicting Lung Cancer outcomes following Radiotherapy: a systematic review. Clin Oncol R Coll Radiol G B.

[15] Zhou C, Hou L, Tang X, Liu C, Meng Y, Jia H (2023). CT-based radiomics nomogram may predict who can benefit from adaptive radiotherapy in patients with local advanced-NSCLC patients. Radiother Oncol J Eur Soc Ther Radiol Oncol.

[16] Wu W, Pierce LA, Zhang Y, Pipavath SNJ, Randolph TW, Lastwika KJ (2019). Comparison of prediction models with radiological semantic features and radiomics in Lung cancer diagnosis of the pulmonary nodules: a case-control study. Eur Radiol.

[17] Zhang L, Chen B, Liu X, Song J, Fang M, Hu C (2018). Quantitative biomarkers for prediction of epidermal growth factor receptor mutation in Non-small Cell Lung Cancer. Transl Oncol.

[18] Nguyen HS, Ho DKN, Nguyen NN, Tran HM, Tam K-W, Le NQK (2023). Predicting EGFR Mutation Status in Non-small Cell Lung Cancer using Artificial Intelligence: a systematic review and Meta-analysis. Acad Radiol.

[19] Park H, Qin L, Guerra P, Bay C, Shinagare A. Decoding incidental ovarian lesions: use of texture analysis and machine learning for characterization and detection of malignancy. Abdom Radiol. 2021;46. 10.1007/s00261-020-02668-3 32728871

[20] Chen C, Zheng A, Ou X, Wang J, Ma X (2020). Comparison of Radiomics-based machine-learning classifiers in diagnosis of Glioblastoma from Primary Central Nervous System Lymphoma. Front Oncol.

[21] Sigel CS, Rudomina DE, Sima CS, Rekhtman N, Travis WD, Geisinger KR (2012). Predicting pulmonary adenocarcinoma outcome based on a cytology grading system. Cancer Cytopathol.

[22] Huang K-Y, Ko P-Z, Yao C-W, Hsu C-N, Fang H-Y, Tu C-Y (2017). Inaccuracy of lung adenocarcinoma subtyping using preoperative biopsy specimens. J Thorac Cardiovasc Surg.

[23] Wu W, Parmar C, Grossmann P, Quackenbush J, Lambin P, Bussink J (2016). Exploratory study to identify Radiomics Classifiers for Lung Cancer Histology. Front Oncol.

[24] Xu Y, Ji W, Hou L, Lin S, Shi Y, Zhou C (2021). Enhanced CT-Based Radiomics to predict Micropapillary Pattern within Lung Invasive Adenocarcinoma. Front Oncol.

[25] He B, Song Y, Wang L, Wang T, She Y, Hou L (2021). A machine learning-based prediction of the micropapillary/solid growth pattern in invasive lung adenocarcinoma with radiomics. Transl Lung Cancer Res.

[26] Li M, Ruan Y, Feng Z, Sun F, Wang M, Zhang L. Preoperative CT-Based Radiomics Combined With Nodule Type to Predict the Micropapillary Pattern in Lung Adenocarcinoma of Size 2 cm or Less: A Multicenter Study. Front Oncol [Internet]. 2021 [cited 2023 Jul 2];11. Available from: https://www.frontiersin.org/articles/10.3389/fonc.2021.788424. 10.3389/fonc.2021.788424 PMC8674565 34926304

[27] Wang F, Wang C-L, Yi Y-Q, Zhang T, Zhong Y, Zhu J-J (2023). Comparison and fusion prediction model for lung adenocarcinoma with micropapillary and solid pattern using clinicoradiographic, radiomics and deep learning features. Sci Rep.

[28] Park S, Lee SM, Noh HN, Hwang HJ, Kim S, Do K-H (2020). Differentiation of predominant subtypes of lung adenocarcinoma using a quantitative radiomics approach on CT. Eur Radiol.

[29] Miao Y, Zhang J, Zou J, Zhu Q, Lv T, Song Y (2017). Correlation in histological subtypes with high resolution computed tomography signatures of early stage lung adenocarcinoma. Transl Lung Cancer Res.

[30] Lederlin M, Puderbach M, Muley T, Schnabel PA, Stenzinger A, Kauczor H-U (2013). Correlation of radio- and histomorphological pattern of pulmonary adenocarcinoma. Eur Respir J.

[31] Lambin P, Leijenaar RTH, Deist TM, Peerlings J, de Jong EEC, van Timmeren J (2017). Radiomics: the bridge between medical imaging and personalized medicine. Nat Rev Clin Oncol.

[32] Le VH, Kha QH, Minh TNT, Nguyen VH, Le VL, Le NQK (2023). Development and validation of CT-Based Radiomics Signature for overall survival prediction in multi-organ Cancer. J Digit Imaging.

[33] Zhang Z (2016). Multiple imputation with multivariate imputation by chained equation (MICE) package. Ann Transl Med.

[34] Bi H, Yin L, Fang W, Song S, Wu S, Shen J. Association of CEA, NSE, CYFRA 21 – 1, SCC-Ag, and ProGRP with clinicopathological characteristics and chemotherapeutic outcomes of Lung Cancer. Lab Med. 2022;lmac122. 10.1093/labmed/lmac122 36282321

[35] Gong J, Liu J, Jiang Y, Sun X, Zheng B, Nie S (2018). Fusion of quantitative imaging features and serum biomarkers to improve performance of computer-aided diagnosis scheme for Lung cancer: a preliminary study. Med Phys.

[36] Obuchi T, Kabashima Y (2016). Cross validation in LASSO and its acceleration. J Stat Mech Theory Exp.

[37] Mayne GC, Woodman RJ, Watson DI, Bright T, Gan S, Lord RV (2023). A method for increasing the robustness of stable feature selection for Biomarker Discovery in Molecular Medicine developed using serum small extracellular vesicle Associated miRNAs and the Barrett’s Oesophagus Disease Spectrum. Int J Mol Sci.

