# Supplementary material for: Predicting micropapillary or solid pattern of lung adenocarcinoma with CT-based radiomics, conventional radiographic and clinical features
Source: Respir Res. 2023 Nov 14;24:282. doi: 10.1186/s12931-023-02592-2 (PMC10647174; doi:10.1186/s12931-023-02592-2)
Supplement: Supplementary file 1 — Supplementary Material 1 [file 12931_2023_2592_MOESM1_ESM.docx]

Table 1 clinical model parameters

| term | estimate | std.error | statistic | p.value |
| --- | --- | --- | --- | --- |
| (Intercept) | -3.11127016 | 0.763473207 | -4.075153 | 4.598409e-05 |
| solid | 3.01462444 | 0.752833265 | 4.004372 | 6.218245e-05 |
| lobulation | -0.75081536 | 0.301714237 | -2.488498 | 1.282839e-02 |
| Air bronchus | -0.94109285 | 0.371960778 | -2.530086 | 1.140345e-02 |
| max | 0.02300164 | 0.008605238 | 2.672981 | 7.518051e-03 |
